# Lung Organoids from hiPSCs Can Be Efficiently Transduced by Recombinant Adeno-Associated Viral and Adenoviral Vectors

**DOI:** 10.3390/biomedicines13040879

**Published:** 2025-04-04

**Authors:** Lyubava Belova, Anna Demchenko, Anastasia Erofeeva, Konstantin Kochergin-Nikitsky, Olga Zubkova, Olga Popova, Tatiana Ozharovskaia, Diana Salikhova, Anna Efremova, Alexander Lavrov, Svetlana Smirnikhina

**Affiliations:** 1 Laboratory of Genome Editing, Research Centre for Medical Genetics, Moscow 115478, Russia; bel.lyubava@gmail.com (L.B.); demchenkoann@yandex.ru (A.D.); anastasija_erofeeva@mail.ru (A.E.); kochnik.ks@gmail.com (K.K.-N.); alexandervlavrov@gmail.com (A.L.); 2 Department of Genetics and Molecular Biology of Bacteria, Gamalei Institute of Epidemiology and Microbiology, Moscow 123098, Russia; olga-zubkova@yandex.ru (O.Z.); t.ozh@yandex.ru (T.O.); 3 Stem Cell Genetics Laboratory, Research Centre for Medical Genetics, Moscow 115478, Russia; diana_salikhova@bk.ru (D.S.); anna.efremova.83@gmail.com (A.E.)

**Keywords:** lung organoids, adeno-associated viral vectors, adenoviral vector, cystic fibrosis, airway basal cells, induced pluripotent stem cells

## Abstract

**Background:** Organoids are a valuable model for studying hereditary diseases such as cystic fibrosis (CF). Recombinant adenoviral (rAdV) and adeno-associated viral (rAAV) vectors are promising tools for CF gene therapy and genome editing. **Objective:** This study aims to determine the most efficient viral vector (rAdV5, rAAV serotypes 5, 6 and 9) and transduction protocol for delivering transgenes to lung organoids (LOs), providing a foundation for future CF gene therapy development. **Methods:** Three transduction protocols were used taking into account the specificities of LOs’ cultivation in specific matrices, both with and without organoid extraction from the matrix. This work was carried out on organoids from a healthy donor (LOs-WT) and on a patient with cystic fibrosis (LOs-CF). **Results:** High transduction efficiency was observed with rAdV5 (30% cells), rAAV6 (>80% cells), and rAAV9 (>40% cells). rAdV5 and rAAV9 transduced basal and secretory cells with >90% efficiency. For rAAV9, Protocol 1 (without extraction of organoids from the matrix) showed lower transduction efficiency (33% for LOs-WT, 9% for LOs-CF), significantly lower than that of Protocols 2 (60% for LOs-WT, 59% for LOs-CF) and 3 (46% for LOs-WT, 35% for LOs-CF) with organoid extraction from the matrix (*p* < 0.005). **Conclusions:** rAdV5 and rAAV9 are the most promising vectors for the delivery of transgenes to basal and secretory cells in a lung organoid model, providing a solid foundation for CF gene therapy development.

## 1. Introduction

Cystic fibrosis (CF) is a severe hereditary disease that is inherited in an autosomal recessive manner and is caused by mutations in the *CFTR* gene [1]. Due to the clear monogenic nature of the disease, since 1994, repeated attempts to develop a gene therapy for this disease have been made. Despite the efforts of many scientific groups around the world, none of the drugs for gene therapy under development have received approval for clinical use. Delivery to airway epithelial cells of the wild-type copy of *CFTR* cDNA by viral or non-viral methods was the only approach for tested gene therapy. This approach is characterized by relative simplicity; however, the effectiveness of such a treatment was short-term or absent, which was associated with the loss of the transgene during the renewal of airway cells [2].

The advent of new techniques for genome manipulation, such as CRISPR-Cas9 [3], opens up new possibilities in the field of gene therapy for cystic fibrosis. For treatment, it is necessary to deliver genetic constructs into cells with a genome editor, which can correct mutations that lead to certain hereditary diseases in a few days (usually 2–3 days). Meanwhile, the correction of the mutation in the *CFTR* gene in epithelial cells in CF patients will be a temporary solution since the therapeutic effect will fade with the self-renewal of cells. In this regard, the basal cells of the lung should be considered the main target for the correction of a mutation in the case of cystic fibrosis because they are the source of all other types of lung cells, including CFTR-expressing epithelial cells [4].

The delivery of any transgene in vivo is always a challenge; so, the choice of the most optimal method is the first task in the development of gene therapy for any disease. Viral vectors based on adenovirus (AdV) and adeno-associated virus (AAV) have a number of advantages over other types of viral vectors and over non-viral delivery methods. Both types of vectors have a natural tropism for respiratory tract cells [5,6] and high efficiency [7]; AdV has a fairly high packing capacity [8], and AAV has low immunogenicity [9]. All these advantages make both types of vectors promising for gene therapy of cystic fibrosis based on genome editing.

In vitro experiment on cell cultures is an integral stage in the development of gene therapy for hereditary diseases. For this purpose, the most optimal is the use of organoids derived from biopsy material or stem cells of patients [10] since they are more closely related to the physiological conditions and functioning of the target organ, unlike cell lines. Organoids are three-dimensional heterogeneous cellular structures cultured in vitro capable of reproducing the functionality and expression profile of the original tissues [11]. Thus, organoids are a promising pre-clinical model for experimental work in gene therapy and genome editing. To date, methods have been developed for obtaining a range of organoids from human-induced pluripotent stem cells (hiPSC) such as lung organoids (LOs) [12,13]. Despite the evident promise of organoids as a model for preclinical research, the question of the applicability of organoid-derived results to the development of viral gene therapy for patients with genetic lung diseases, such as CF, remains unresolved. The transduction of organoids and patient tissues by viral vectors differs significantly due to several factors. The structure and microenvironment of organoid cultures differ from those of native lung tissue [14]. While LOs are cultured in matrices containing extracellular matrix proteins and growth factors, the high viscosity of these components has been shown to impede the penetration of viral particles into organoid cells [9]. In addition to this, in vivo barriers such as mucociliary clearance and immune response have the capacity to further reduce delivery efficiency. Furthermore, the duration of the therapeutic effect in organoids may not fully reflect clinical realities. A significant limitation of gene therapy for CF is the loss of the therapeutic gene due to the renewal of airway epithelial cells. Therefore, the most crucial aspect is the delivery of therapeutic genes to basal cells as they serve as progenitors for airway epithelial cells and can ensure a more sustained therapeutic effect [15]. Therefore, the data obtained on the transduction of organoids with different viral serotypes are of primary importance for the study of transduction mechanisms and the assessment of the specificity of viral vectors for lung epithelial cells. They assist in the identification of the most promising serotypes for further study and reveal the molecular factors affecting transduction efficiency. However, in order to evaluate clinical applicability, it is necessary to perform additional in vivo experiments to simulate the real conditions of genetic material delivery to lung tissues. The employment of organoids constitutes a significant advancement in the realm of scientific inquiry, particularly in the domains of viral transduction and gene editing research. Nevertheless, it is imperative to acknowledge the constraints inherent in this model when formulating efficacious therapeutic strategies.

By the end of 2022, a number of studies for transduction of different types of organoids by viral vectors rAAV [16,17,18,19,20,21,22,23,24] and rAdV [25,26,27] had been published. However, there was limited information available regarding the transduction of LOs [22]. The main difficulty in working with organoids, in particular, with transduction, is the cultivation of organoids in special matrices to maintain their 3D structure. Such matrices support the vitality of organoids by containing intracellular matrix proteins of the basal membrane, among which are proteoglycans such as heparan sulfate proteoglycan (HSPG), and various growth factors, which are among the receptors for some serotypes of AAV [9] and AdV [28]. High viscosity and protein availability reduce the rate of passage of viral vectors, as well as the efficiency of organoid transduction [9]. The objective of this study was to assess the effectiveness of transducing lung organoids, derived from iPSCs, using various serotypes of adenoviral and adeno-associated viral vectors.

Here, we performed transduction of LOs differentiated from hiPSC from a healthy donor (*wt*/*wt*) (LOs-WT) and from a cystic fibrosis patient with homozygous F508del mutation of the *CFTR* gene (F508del/F508del) (LOs-CF) by recombinant viral vectors with tropism to human respiratory cells—rAdV serotype 5 and rAAVs vectors serotypes 5, 6 and 9—with genes of fluorescent reporter.

## 2. Materials and Methods

### 2.1. Generation of Lung Organoids

Human lung organoids were generated from human induced pluripotent stem cells (hiPSCs) lines from a healthy donor (*wt*/*wt*) [29] and from a cystic fibrosis patient with homozygous F508del mutation of the *CFTR* gene (F508del/F508del) [30]. The hiPSC lines were deposited at the Moscow Branch of the Biobank “All-Russian Collection of Biological Samples of Hereditary Diseases” (Research Centre for Medical Genetics, Moscow, Russia). This study was approved by the Ethics Committee of the Research Centre for Medical Genetics (Moscow, Russia) and conducted in accordance with the provisions of the Declaration of Helsinki (1975).

Prior to differentiation, the hiPSCs lines were maintained under feeder-free conditions in 6-well tissue culture dishes (Costar, cat. No. 3506) coated with the Vitronectin (VTN-N) Recombinant Human Protein (Thermo Fisher Scientific, Waltham, MA, USA) in the mTeSR1 medium (Stem Cell Technologies, Vancouver, BC, Canada) for 14 days. Differentiation of hiPSCs into lung organoids was performed as described in this article, previously published by our research group [31].

### 2.2. Generation of Recombinant AAV Vectors

Plasmid pAAV-CMV-GFP was a gift from Dr. S.P. Chumakov from the Institute of Bioorganic Chemistry (Moscow, Russia); pAAV2/9n was a gift from James M. Wilson (Addgene plasmid #112865; http://n2t.net/addgene:112865 (accessed on 19 March 2025); RRID:Addgene 112865); pAAV2/5 was a gift from Melina Fan (Addgene plasmid #104964; http://n2t.net/addgene:104964 (accessed on 19 March 2025); RRID:Addgene_104964); pAdDeltaF6 was a gift from James M. Wilson (Addgene plasmid #112867; http://n2t.net/addgene:112867 (accessed on 19 March 2025); RRID:Addgene_112867). Plasmid pAAV2/6 was purchased from TakaraBio (#6651, Kusatsu, Shiga, Japan). Triple plasmid transfection (pAAV-CMV-GFP, pAdDeltaF6 and one of the AAV packaging plasmid—pAAV2/9n or pAAV2/5 or pAAV2/6) of HEK293T cells at 90% confluence was performed with an equimolar ratio of the plasmids used. Transfection was carried out in twelve 150 mm cultural plates with 5 mg/mL of transfection agent linear polyethylenimine (PEI 25 kDa MW, Polysciences, Warrington, PA, USA) [32]. Three days after transfection, the cells and medium were harvested and monomeric viral vectors were concentrated and purified. Virus-containing cell media were processed through PEG precipitation (PEG 8000 10%, overnight incubation). Cell pellets were lysed in cell lysis buffer (50 mM TrisCl pH8, 150 mM NaCl) using multiple freeze/thaw circles. The solution with purified virus was further combined with PEG precipitated virus from cell media and treated by Benzonase (Sigma-Aldrich, St. Louis, MI, USA) (50 units per ml). The vectors were then purified by sedimentation through the layer of 20% sucrose (160,000 g, 120 min, 10 °C). Genome titers of the acquired vectors preparations were analyzed by qPCR with ITR2-specific primers (forward: 5′-GGAACCCCTAGTGATGGAGTT; reverse: 5′-CGGCCTCAGTGAGCGA) [33].

### 2.3. Generation of a Recombinant Adenoviral Vector

The recombinant plasmid pAdV5-mCherry containing a full Ad5 genome with deleted E1 and E3 regions and a red fluorescent protein (mCherry) reporter gene under the control of a cytomegalovirus (CMV) immediate early promoter was generated by homologous recombination in Escherichia coli strain BJ5183 according to the standard protocol. Briefly, E. coli cells were co-transformed with two plasmids: pAd5dE1/E3 and pShuttle-CMV-mCherry in order to obtain the plasmid with a full-size genome AdV5 and a red fluorescent protein gene (pAdV5-mCherry). Plasmid pAd5dE1/E3 was obtained earlier using a standard method for adenovirus genome modification. Wild-type Ad5 virus strain was obtained from the State Collection of Viruses (FSBI “National Research Centre for Epidemiology and Microbiology named after the honorary academician N.F. Gamaleya” of the Ministry of Health of the Russian Federation). The nucleotide sequence of Ad5 was determined via full-genome sequencing. To construct the shuttle plasmid (pShuttle-CMV), plasmids containing the left (1–342 bp and 3534–3646 bp) and the right homology arms (35,276–35,938 bp) were chemically synthesized by Evrogen JSC (Moscow, Russia). Expression cassette was placed between 342 and 3534 bp of the left homology arm. The construction of backbone plasmid pAd5dE1 was achieved by homologous recombination of the linearized pShuttle-CMV with the genome DNA of Ad5. Using standard genetic engineering methods, the E3 region of the adenovirus genome (from 28,134 to 30,818 bp) was removed from the constructed plasmid pAd5dlE1, resulting in the final plasmid pAd5dE1/E3. The reporter gene mCherry was chemically synthesized by Evrogen JSC (Moscow, Russia) and cloned into pShuttle-CMV. Prior to the transformation, pAd5dE1/E3 and pShuttle-CMV-mCherry were linearized by PacI and PmeI, respectively. Recombinant clones were analyzed using polymerase chain reaction (PCR) and restriction assay.

The recombinant AdV5-mCherry virus was obtained via lipofection of the PacI-linearized pAdV5-mCherry plasmid into HEK293 cells using Lipofectamine 2000 (Thermo Fisher Scientific, Waltham, MA, USA), according to the product’s protocol. The identity of the virus was verified by PCR. To grow the virus HEK293, the cells were seeded into ten 150 mm Petri dishes and incubated overnight to 80% confluence, after which these were infected with AdV5-mCherry. When full CPE occurred, recombinant AdV5-mCherry was purified by double-cesium chloride gradient centrifugation, with the purity and identity of the AdV5 verified by PCR. Titer was determined using a plaque assay on the HEK293 cell culture.

### 2.4. rAAV and rAdV5 Infection and Measurement of GFP and mCherry-Positive Cells

LOs’ transduction was performed according to three transduction protocols: Protocol 1—organoids’ transduction by viral vectors directly through the matrix layer; Protocol 2—transduction of organoids extracted from the matrix by viral vectors followed by insertion into the matrix; Protocol 3—transduction of organoids extracted from the matrix with additional incubation time within 2 h at 37C in an Orbital Shaker-Incubator (BioSan ES-20, Riga, Latvia) at a rate of 250 rpm, followed by insertion into the matrix. Transduction of organoids was performed at 22 days from the beginning of differentiation. All viral vectors were tittered with multiplicity of infection (MOI) 1 × 10^9^ and 1 × 10^10^ vg/organoid for rAAV-GFP, and 4 × 10^6^ and 8 × 10^6^ PFU (plaque-forming unit) for 60 organoids for rAdV5-mCherry. The chosen MOI and PFU were used in all down-stream transduction experiments. All experiments were performed in three biological and three technical replicates. Transduction efficiencies were assessed at 6 days post infections using flow cytometry.

### 2.5. Immunofluorescence Staining

Immunostaining of lung organoids after transduction of rAAV-GFP 6 and 9 serotypes, and rAdV5-mCherry was performed according to the protocols of Dekkers et al. [34]. Briefly, droplets with organoids were mechanically dislodged, centrifuged for 5 s at 6300× *g*, and fixed in a chilled 4% formalin solution for 45 min at +4 °C. Then, the organoids were permeabilized in a cold solution of 0.1% Tween 20 (Merck, Kenilworth, NJ, USA) for 10 min at +4 °C and centrifuged for 5 min at 70× *g* at +4 °C; the precipitate was blocked with a cold solution of 0.1% Triton X-100 and 0.2% BSA in DPBS without Ca^2+^ and Mg^2+^ for 15 min at +4 °C. Then, a solution of primary antibodies TP63 (Thermo Fisher Scientific, Waltham, MA, USA, cat. No. 703809), SCGB3A2 (Abcam, Cambridge, UK, cat. No. ab181853), Anti-GFP antibody [9F9.F9] (Abcam, Cambridge, UK, cat. No. ab1218) for organoids after AAV transduction and Anti-mCherry antibody [1C51] (Abcam, Cambridge, UK, cat. No. ab125096) for organoids after AdV transduction was added into 0.1% Triton X-100 and 0.2% BSA in DPBS without Ca^2+^ and Mg^2+^, and the mixture was incubated overnight at +4 °C. The organoids were washed twice with a solution of 0.1% Triton X-100 and 0.2% BSA in DPBS without Ca^2+^ and Mg^2+^ for 2 h at +4 °C. Then, a solution of the secondary antibodies Goat Anti-Mouse IgG H&L (Alexa Fluor^®^ 488) (Abcam, Cambridge, UK, cat. No. ab150113) and Goat Anti-Rabbit IgG (H + L) (Alexa Fluor™ 594) (Thermo Fisher Scientific, Waltham, MA, USA, cat. No. A-11037) was added into 0.1% Triton X-100 and 0.2% BSA in DPBS without Ca^2^+ and Mg^2+^, and the mixture was incubated overnight at +4 °C. The organoids were washed twice with a solution of 0.1% Triton X-100 and 0.2% BSA in DPBS without Ca^2+^ and Mg^2+^ for 2 h at +4 °C. After that, the organoids were stained with DAPI (Abcam, Cambridge, UK) for 10 min at room temperature and subsequently centrifuged for 5 s at 6300× *g*; the pellet was resuspended in a solution of 2.5 mM fructose (Sigma Aldrich, St. Louis, MI, USA) in 60% glycerol (PanReac Applichem, Chicago, IL, USA) and incubated for 20 min at room temperature. The suspension was transferred onto a glass slide (Pyrex (Corning), New York, NY, USA) and covered with a cover glass (Pyrex (Corning), New York, NY, USA); microscopy was performed on a TCS SP8 confocal laser scanning microscope (Leica Microsystems, Wetzlar, Germany).

Immunostaining of the iPSC (Supplementary) line from a healthy donor allowed us to confirm SCGB3A2 and TP63 primary antibody specificity. For immunostaining of iPSCs, the cells were grown on a Matrigel-coated 48-well plate. The cells were washed twice with DPBS (PanEco, Russia) and fixed with 4% PFA (Carl Roth, Germany) in PBS for 10 min at 37 °C. The cells were permeabilized in a cold solution of 0.1% Tween 20 (Merck, Darmstadt, Germany) for 10 min at +4 °C and washed three times with DPBS; then, the cells were blocked with a cold solution of 0.1% Triton X-100 (Helicon, Moscow, Russia) and 0.2% BSA (Sigma Aldrich, USA) in DPBS for 30 min at room temperature (RT: 20 to 25 °C). Primary antibodies TP63 (Thermo Fisher Scientific, Waltham, MA, USA, cat. No. 703809) and SCGB3A2 (Abcam, Cambridge, UK, cat. No. ab181853) were added and incubated for 1 h at RT. Following this, the cells were washed three times with DPBS. Then, secondary antibodies Goat Anti-Rabbit IgG (Alexa Fluor^®^ 488) (Absin, Shanghai, China, cat. No. abs20025) were added and incubated for 30 min at RT; the cells were washed three times with DPBS. After that, the cells were stained with DAPI (Abcam, UK) and visualized using the Lionheart FX Automated Microscope.

### 2.6. Flow Cytometry Analysis

The flow cytometry analysis was performed six days post infection. For the analysis, organoids were collected and mechanically broken using a syringe, then disaggregated using Trypsin-EDTA solution (Paneco, Moscow, Russia) for 15 min and washed PBS without Mg^2+^ and Ca^2+^. The cells were then stained with 7-Aminoactinomycin D (7-AAD) (Thermo Fisher Scientific, Waltham, MA, USA) to allow for detection of live cells. The cells were counted by flow cytometry using CytoFLEX S V2-B2-Y2-R0 Flow Cytometer and CytExpert Software 2.4 (all Beckman Coulter, Brea, CA, USA). The cells were firstly gated based on their forward scatter area (FSC-A) and side scatter area (SSC-A) properties and then single cells were gated based on FSC-A and forward scatter height (FSC-H) properties. The blue (488 nm) laser was used to excite the 7-AAD and fluorescence was captured using the red (647 nm) filter channels. The blue (488 nm) laser was used to excite the GFP, and fluorescence staining was captured using the green (525 nm) filter channel. The yellow (587 nm) laser was used to excite the mCherry, and fluorescence staining was captured using the red (610 nm) filter channel.

### 2.7. Statistical Analysis

Statistical analysis of the data was performed by GraphPad Prism v.9.1.1. Transduction efficiency was assessed using a one-way ANOVA with Dunnett’s T3 multiple comparisons test. Differences were considered significant at *p* < 0.05.

## 3. Results

### 3.1. Transduction of LOs Using rAAV Vectors Serotypes 5, 6 and 9

**Figure 1:**
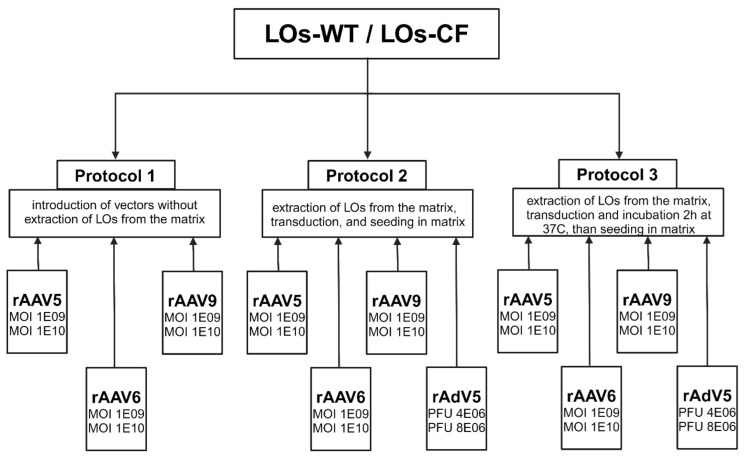
Scheme of LOs transduction using rAAV and rAdV vectors.

On the 22nd day of differentiation, transduction of LOs obtained from a healthy donor (LOs-WT) and a patient with cystic fibrosis (LOs-CF) was carried out using three protocols for introducing viral vectors (Figure 1). Protocol 1 involved introducing a viral vector without extracting organoids from the matrix layer, allowing for the assessment of the impact of viral vectors on the basolateral surface of lung epithelial cells. In Protocols 2 and 3, the organoids were removed from the matrix, disrupted, transduced with viral vectors, and then transferred back to the matrix. Transduction of destroyed organoids allowed for mimicking gene transfer to both the basolateral and apical surfaces of lung epithelial cells. Protocol 3 differed in the additional incubation time of organoids with the viral vector for two hours. The green fluorescent protein (GFP) gene was used as a reporter in all AAV serotypes. LOs were transduced at MOI values of 1 × 10^9^ and 1 × 10^10^ vg/organoid for rAAV-GFP. We also applied an MOI of 1 × 10^8^ for rAAV6 and rAAV9 preparations (Figures S1 and S2); however, we obtained low transduction efficiency values. In the case of the rAAV5 vector, we did not use doses below 1 × 10^9^ due to low transduction efficiency (Figure S3). The fluorescent signal was recorded 72 h after transduction, and after six days, the transduction efficiency was analyzed by flow cytometry. The cell death rate during the analysis was no higher than 5%.

**Figure 2:**
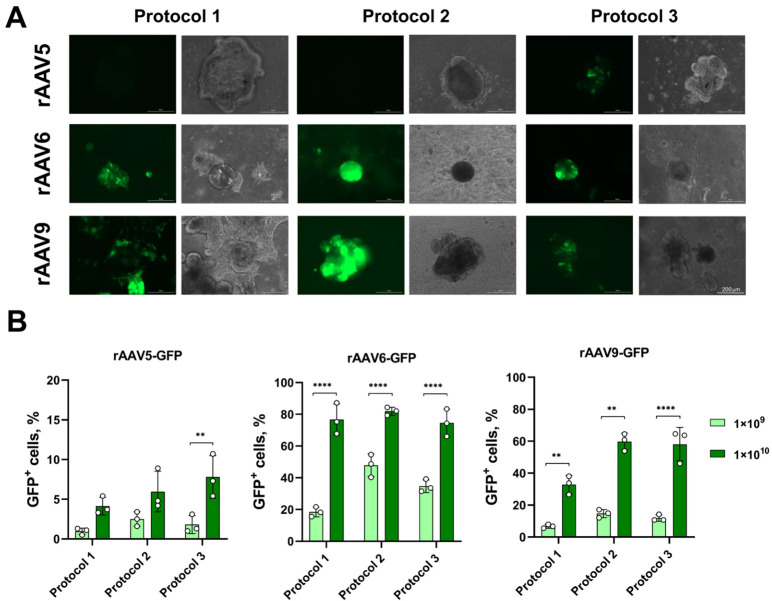
Efficiency of LOs-WT transduction using rAAV vectors. (**A**) Representative image of GFP fluorescence in LOs-WT on day six after transduction using rAAV5, 6 and 9 at MOI 1 × 10^10^ vg/organoid (Complex Lionheart FX, Magnification ×10). (**B**) rAAV dose-dependent transduction efficiency of LOs-WT using three different protocols of transduction. Data are presented as mean value ± SD; the differences were considered statistically significant when *p* value < 0.005 (**), *p* value < 0.0001 (****) using Dunnett’s T3 multiple comparison test.

**Figure 3:**
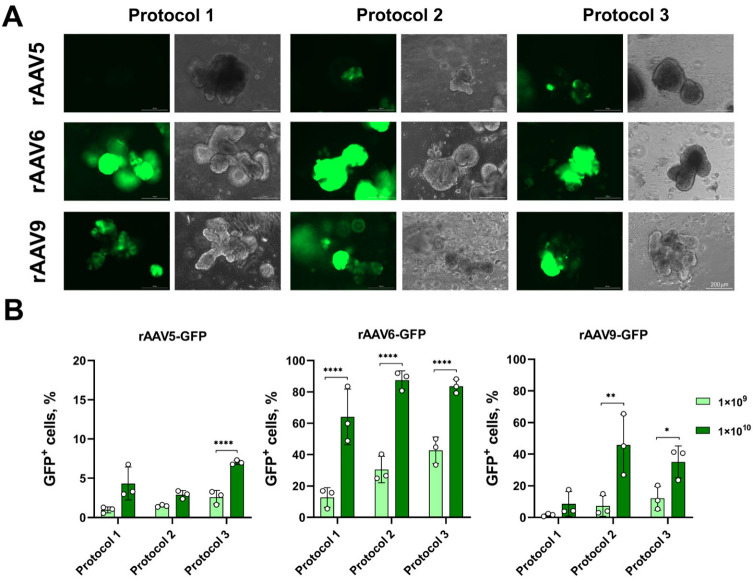
Transduction efficiency of LOs-CF using three different infection protocols. (**A**) Representative image of LOs-CF on day six after transduction using rAAV vectors at MOI 1 × 10^10^ vg/organoid (Complex Lionheart FX, Magnification ×10). (**B**) GFP expression in LOs-CF after rAAV transduction using three infection protocols. Data are presented as mean value ± SD; the differences were considered statistically significant when *p* value < 0.05 (*), *p* value < 0.005 (**), *p* value < 0.0001 (****) using Dunnett’s T3 multiple comparison test.

The highest transduction efficiency for both LOs-WT and LOs-CF was observed with rAAV6-GFP and rAAV9-GFP (Figure 2 and Figure 3). Therefore, when transducing LOs-WT, the maximum number of GFP^+^ cells was achieved using rAAV6, with approximately 80% of GFP^+^ cells at MOI 1 × 10^10^ for all three transduction protocols. For rAAV9, the maximum number was about 60% of GFP^+^ cells at MOI 1 × 10^10^, which was observed using Protocols 2 and 3, and 33% in the case of Protocol 1 (Figure 2B). For LOs-CF, the highest efficacy was obtained using Protocols 2 and 3 for both serotypes: 88% and 84% GFP^+^ cells at MOI 1 × 10^10^ for rAAV6-GFP, and 46% and 35% GFP^+^ cells at MOI 1 × 10^10^ for rAAV9-GFP, respectively (Figure 3B). The minimum efficiency among all viral preparations was shown with rAAV5-GFP, not exceeding 8% at MOI 1 × 10^10^ (Figure 2 and Figure 3).

**Figure 4:**
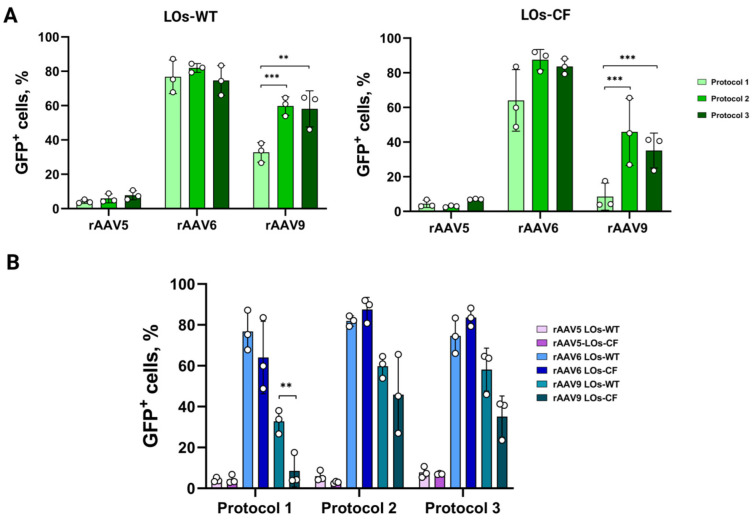
Comparison of LOs-WT and LOs-CF transduction efficiency by rAAV vectors using three infection protocols. (**A**) The graphs provide a comparison of the efficiency of transduction protocols for LOs for each rAAV vector of serotypes 5, 6, and 9. (**B**) The present study compares the efficiency of transduction protocols for LOs-WT and LOs-CF using rAAV viral vectors. The data presented were obtained at an MOI of 1E10. Data are presented as mean value ± SD; the differences were considered statistically significant when *p* value < 0.005 (**), *p* value < 0.0005 (***) using Dunnett’s T3 multiple comparison test.

It is noteworthy that we did not find any significant differences between the transduction protocols for both LOs-WT and LOs-CF in the case of the rAAV5 and 6 serotype vectors (Figure 2 and Figure 3). A comparison of the efficiency of the transduction protocols for rAAV serotype 9 showed a notably lower efficiency of the protocol without extraction of LOs (Protocol 1) compared to the protocols with extraction of LOs (Protocols 2 and 3) for both LOs-WT and LOs-CF (Figure 4). In addition, we found a significantly higher efficiency of transduction for LOs-WT compared to LOs-CF in the case of Protocol 1 (Figure 4).

Thus, we have demonstrated the high efficiency of transducing LOs with rAAV serotypes 6 and 9 using three transduction protocols.

### 3.2. Transduction of LOs Using rAdV5 Vector

**Figure 5:**
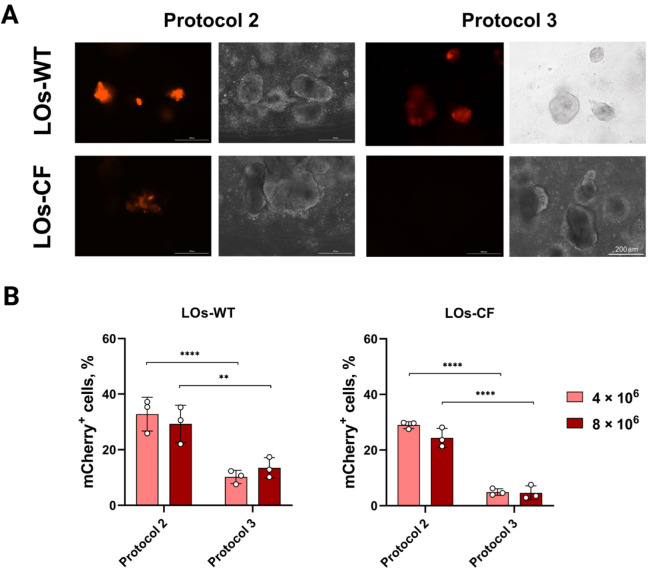
rAdV dose-dependent transduction efficiency of LOs-WT and LOs-CF using two transduction protocols. (**A**) Representative image of LOs-WT and LOs-CF on day six after transduction using rAdV5-mCherry at PFU 4 × 10^6^ (Complex Lionheart FX, Magnification ×10). (**B**) mCherry expression in LOs-WT and LOs-CF after rAdV5 transduction using two infection protocols. Data are presented as mean value ± SD; the differences were considered statistically significant when *p* value < 0.005 (**), *p* value < 0.0001 (****) using Dunnett’s T3 multiple comparison test.

When transducing LOs-WT and LOs-CF with the rAdV5-mCherry viral vector, we used two introduction protocols: Protocol 2 and Protocol 3 (Figure 1). We transduced LOs at PFU values of 2.5 × 10^5^, 5 × 10^5^, 1 × 10^6^, 4 × 10^6^, 8 × 10^6^, and 1.6 × 10^7^ for rAdV5-mCherry per 60 organoids. The dose of 1.6 × 10^7^ PFU was toxic to the organoids, and doses below 4 × 10^6^ PFU showed low transduction efficiency in LOs (Figure S4). Thus, we selected two doses for rAdV, 4 × 10^6^ and 8 × 10^6^ PFU. We recorded the initial fluorescent signal 48 h after transduction, and after six days, we analyzed the transduction efficiency by flow cytometry. According to flow cytometry, we showed the maximum of mCherry^+^ cells at PFU 4 × 10^6^ when transducing both LOs-WT and LOs-CF (31% and 26%, respectively) using Protocol 2 (Figure 5). Notably, when comparing the transduction efficiency using two protocols for the rAdV5-mCherry vector, we observed a significant difference for both LOs-WT and LOs-CF at both PFU values (Figure 5B). However, we did not observe a significant difference in the transduction efficiency of LOs-WT and LOs-CF (Figure 5B).

**Figure 6:**
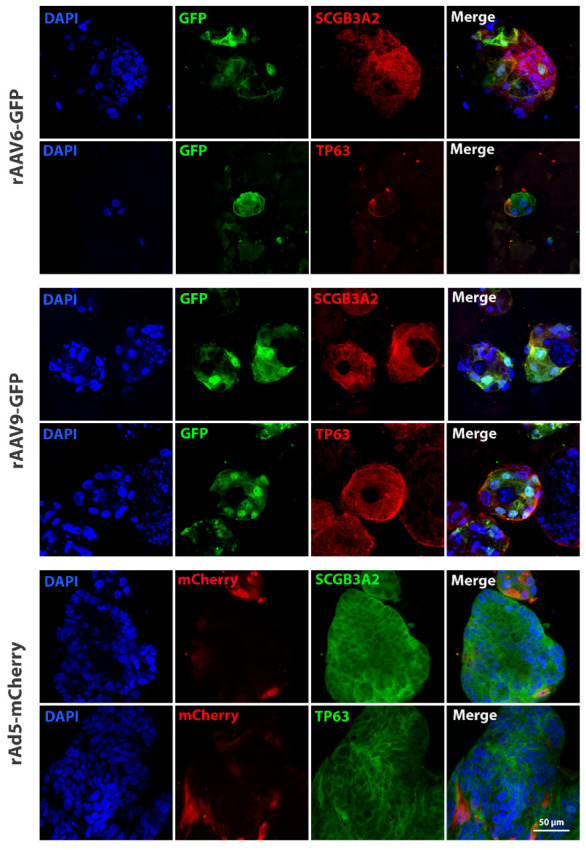
Representative images from confocal microscopy of LOs stained against basal cell marker (TP63), secretory cell marker (SCGB3A2), GFP and mCherry proteins after transduction by rAAV6, rAAV9 and rAdV5 using Protocol 2. Nuclei were stained with DAPI (blue). Magnification ×63.

Taking into account the heterogeneous composition of lung organoids, we aimed to determine which cell types were transduced. We stained LOs-CF transduced by Protocol 2 with vectors rAAV6-GFP (MOI 1 × 10^10^), rAAV9-GFP (MOI 1 × 10^10^), and rAdV5-mCherry (PFU 4 × 10^6^) for two types of cell-specific markers characteristic of secretory cells (SCGB3A2) and basal cells (TP63). Based on the results of immunocytochemistry (ICC) followed by flow cytofluorimetry analysis, we determined that during transduction with the rAAV6-GFP vector, about 30% of SCGB3A2^+^ and 65.5% of TP63^+^ cells were positive for GFP^+^ (successfully transduced). In the case of using the rAAV9-GFP vector, 81% of SCGB3A2^+^ and 92.6% of TP63^+^ were positive for GFP. Maximum transduction efficiency was demonstrated for rAdV5-mCherry, with 91.8% TP63^+^mCherry^+^ and 98.8% SCGB3A2^+^mCherry^+^ (Figure 6) (Table S1). Since the markers TP63 and SCGB3A2 stain positively for the majority of cells in LOs, to confirm their specificity, staining of iPSCs from a healthy donor was performed, in which, as expected, iPSCs did not stain for either marker (Figure S5).

Therefore, we have determined that the rAAV9-GFP and rAdV5-mCherry vectors have a pronounced cellular tropism for the basal and secretory cells of LOs.

The data reveal that transduction of lung organoids by adenoviral vector serotype 5 and AAV vector serotype 9 results in transgene delivery to basal and secretory cells of the lung, with a percentage of 92% and 93%, respectively.

## 4. Discussion

In this study, we have demonstrated that adenoviral vector serotype 5 and AAV vector serotype 9 are effective in transducing lung organoids, specifically targeting basal and secretory cells with an efficiency of 92% and 93%, respectively. Notably, our findings indicate that these vectors transduce basal cells, which are crucial progenitor cells capable of differentiating into various types of lung epithelial cells [35]. This insight is particularly significant as genetic modifications made to basal cells can have lasting effects, providing a foundation for the development of novel gene therapy drugs. The results of our work can be used to develop gene therapies for monogenic lung diseases, such as cystic fibrosis, for which there is currently no gene therapy available.

We selected the viral vectors utilized in this study based on the published literature, which suggests that they possess tropism for respiratory tract epithelial cells [9,36]. However, we rejected the usage of AAV serotype 2 as it has non-selective transduction in many human tissues and organs [37].

Adenoviral vectors have several advantages that make them a potential method for delivering genetic editors to lung cells. They have a large packing capacity, can accommodate all existing modifications in CRISPR-Cas9-based genome editors, including prime editing, do not integrate into the genome, and are relatively easy and cost-effective to produce at an industrial scale [38]. AAV is considered the gold standard of viral delivery for gene therapy since it does not cause insertional mutagenesis or immune responses [9].

The use of organoids as a model for organ-specific monogenic hereditary diseases opens numerous possibilities for the development of gene therapy methods, including genome editing. Lung organoids can survive only in matrices that are heterogeneous complexes of proteoglycans and growth factors. Some of these growth factors are common receptors for most AAV serotypes [5], the binding of which can decrease the efficiency of organoid transduction [9]. For this reason, partial [24,27] and/or complete [23,27] extraction of organoids from the matrices for subsequent organoids transduction by viral vectors was performed to avoid potential binding of viral capsids to the protein components of the matrix.

In addition, we considered that lung organoids contain heterogeneous cell types and an axial orientation of the cells, which is responsible for different presentation of the receptors on their apical and basolateral surfaces. Disruption of organoids enables cells to be transduced through both basolateral and apical surfaces. To assess the efficacy of transgene delivery, we utilized green fluorescent protein (GFP) and mCherry and applied three different methods of viral vector introduction to the organoids. These methods included (1) transducing organoids without extracting them from the matrix, (2) extracting, disrupting, and transducing organoids with viral vectors before reinserting them into the matrix, and (3) extracting, disrupting, and transducing organoids with viral vectors followed by two-hour incubation before reinsertion into the matrix.

We found that the rAAV6 serotype was the most effective vector, resulting in approximately 88% and 84% of GFP^+^ cells for LOs-WT and LOs-CF at MOI 1 × 10^10^. Interestingly, we did not find any significant differences between the protocols. However, when using the rAAV9 vector to transduce LOs, we observed notable differences in efficiency between the protocols. Specifically, Protocol 1 demonstrated a transduction efficiency of 33% for LOs-WT and 9% for LOs-CF, which was significantly lower than the efficiency of Protocols 2 (60% for LOs-WT and 59% for LOs-CF) and 3 (46% for LOs-WT and 35% for LOs-CF) (*p* < 0.005). This discrepancy in transduction efficiency could be attributed to Corning^®^ Matrigel^®^ Matrix’s proteins that bind to the surface proteins of the AAV serotype 9 capsid. However, determining the exact interactions is challenging since the composition of Corning^®^ Matrigel^®^ Matrix remains unknown. Furthermore, new AAV receptor interactions with the cell are still being discovered, making it difficult to establish a definitive explanation.

In the case of LOs transduction with the rAdV5-mCherry viral vector, we observed the maximum efficiency at PFU 4 × 10^6^ using Protocol 2 (31% for LOs-WT and 26% for LOs-CF). However, unlike AAV-based viral vectors, where differences in efficiency were observed between Protocol 1 and protocols involving organoid extraction, with rAdV, we observed a significant difference in efficiency between the two protocols characterized by LO extraction from the matrix and differing in the additional incubation time of organoids with the viral vector (Protocol 2 and Protocol 3) (Figure 4). We posit that the additional incubation time of the vector with organoids outside the matrix could have influenced the rate of virion interaction with organoid cells and increased the viral load on the cells, thereby exhibiting cytopathic effects. Consequently, cells receiving a lower virus dose were able to form organoids, in which, however, the percentage of mCherry^+^ cells did not exceed 14% for LOs-WT and 5% for LOs-CF when assessing transduction efficiency.

Despite the high efficiency of rAAV5 in transgene delivery to respiratory tract cells, as shown in many studies [39,40], our work yielded more modest results with no more than 8% efficiency. This is particularly noteworthy when compared to the high efficiency of other viral vectors.

To date, only one paper has been published on the transduction of lung organoids with AAV-based viral vectors [20]. The authors transduced lung bud organoids using microinjection to target the apical surface of the cells in organoids with several rAAV serotypes. The most effective vectors were found to be rAAV serotype 6 and two mutant vectors based on serotype 6 (rAAV6.2 and rAAV6.2FF). However, transduction of the apical surface of organoids with serotype 9 and 5 vectors did not result in expression of the GFP reporter protein.

## 5. Conclusions

Our work demonstrates that viral vectors based on the rAdV serotype 5 and rAAV serotypes 6 and 9 have a high tropism for lung basal and secretory cells in lung organoids obtained from iPSCs of a healthy donor and a patient with cystic fibrosis. These vectors can be used to deliver various therapeutic genes to develop gene therapy.

## Data Availability

The raw data supporting the conclusion of this article will be made available by the authors without undue reservation.

## Supplementary Materials

The following supporting information can be downloaded at: https://www.mdpi.com/article/10.3390/biomedicines13040879/s1, Figure S1: Flow cytometry analysis of LOs after transduction by rAAV6-GFP at MOI 1 × 10^8^, 1 × 10^9^ and 1 × 10^10^; Figure S2: Flow cytometry analysis of LOs after transduction by rAAV9-GFP at MOI 1 × 10^8^, 1 × 10^9^ and 1 × 10^10^; Figure S3: Flow cytometry analysis of LOs after transduction by rAAV5-GFP at MOI 1 × 10^9^ and 1 × 10^10^; Figure S4: Flow cytometry analysis of LOs after transduction by rAdV5-mCherry at PFU 2.5 × 10^5^, 5 × 10^5^, 1 × 10^6^, 4 × 10^6^, 8 × 10^6^ and 1.6 × 10^7^; Figure S5: Representative images from fluorescent microscopy of iPSCs stained against TP63 and SCGB3A2 markers; Figure S6: Representative confocal microscopy images of Los stained only with the secondary antibody after viral vectors transduction; Table S1: The data of the ICC.

## Abbreviations

The following abbreviations are used in this manuscript:

CF	Cystic fibrosis
CFTR	Cystic fibrosis transmembrane conductance regulator
AdV	Adenovirus
AAV	Adeno-associated virus
hiPSC	Human-induced pluripotent stem cells
LO	Lung organoid
HSPG	Heparan sulfate proteoglycan
MOI	Multiplicity of infection
PFU	Plaque-forming unit
SCGB3A2	Secretoglobin Family 3A, Member 2
TP63	Tumor protein p63
