# Supplementary figures and images for: Lung Organoids from hiPSCs Can Be Efficiently Transduced by Recombinant Adeno-Associated Viral and Adenoviral Vectors
Source: Biomedicines. 2025 Apr 4;13(4):879. doi: 10.3390/biomedicines13040879 (PMC12024971; doi:10.3390/biomedicines13040879)

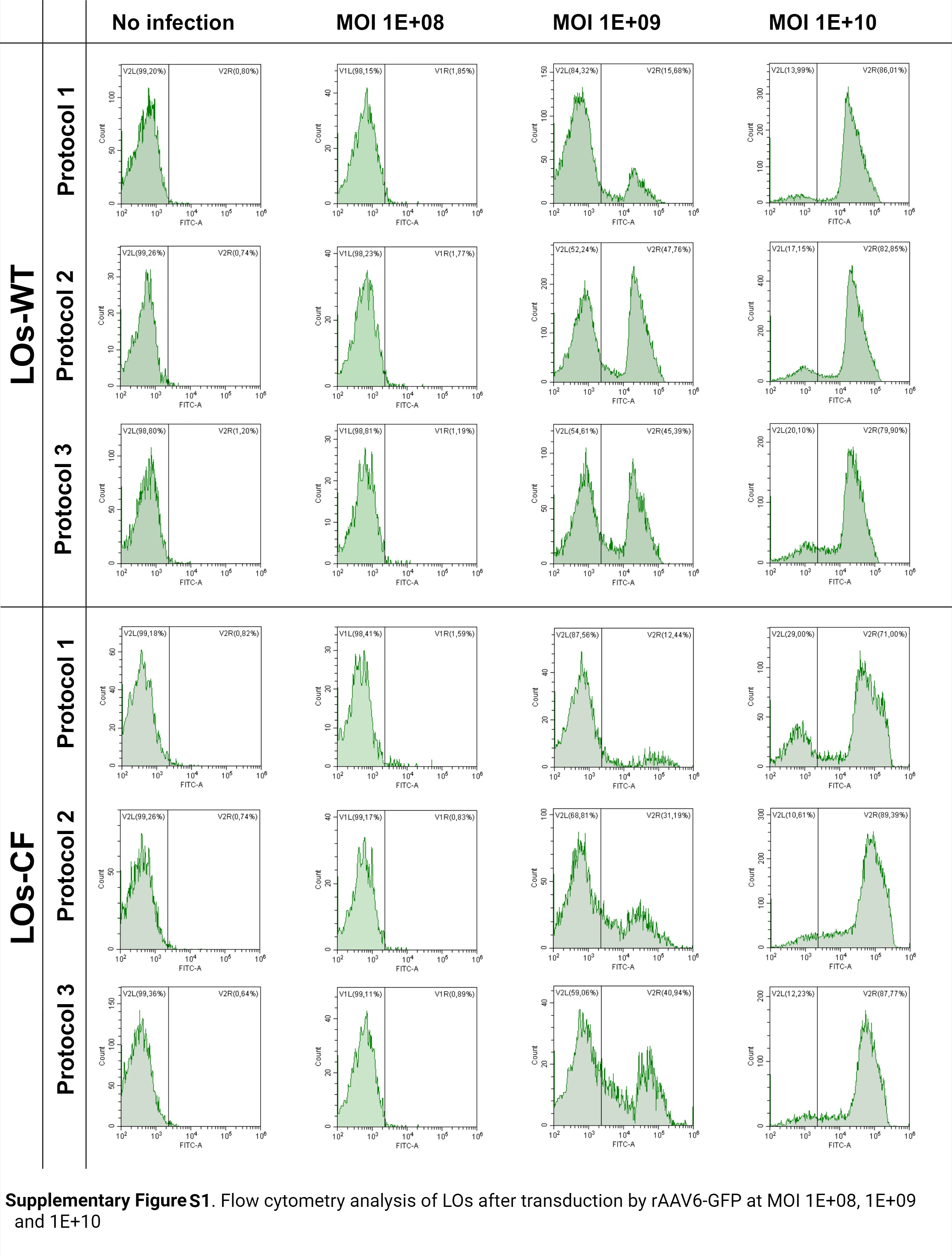

Supplement: Supplementary file 1 [file biomedicines-13-00879-s001.zip › Supplementary Figure S1.jpeg]

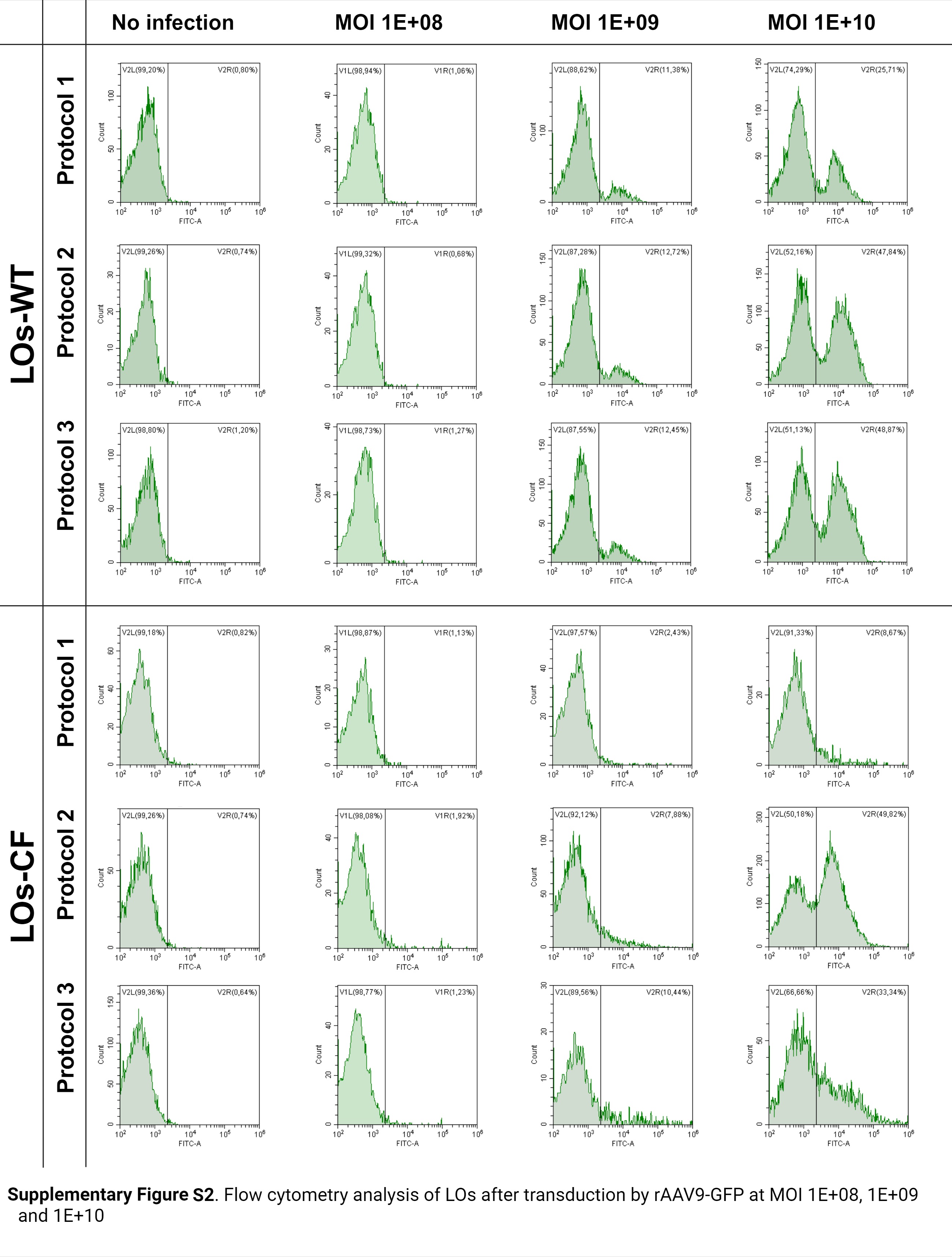

Supplement: Supplementary file 1 [file biomedicines-13-00879-s001.zip › Supplementary Figure S2.jpeg]

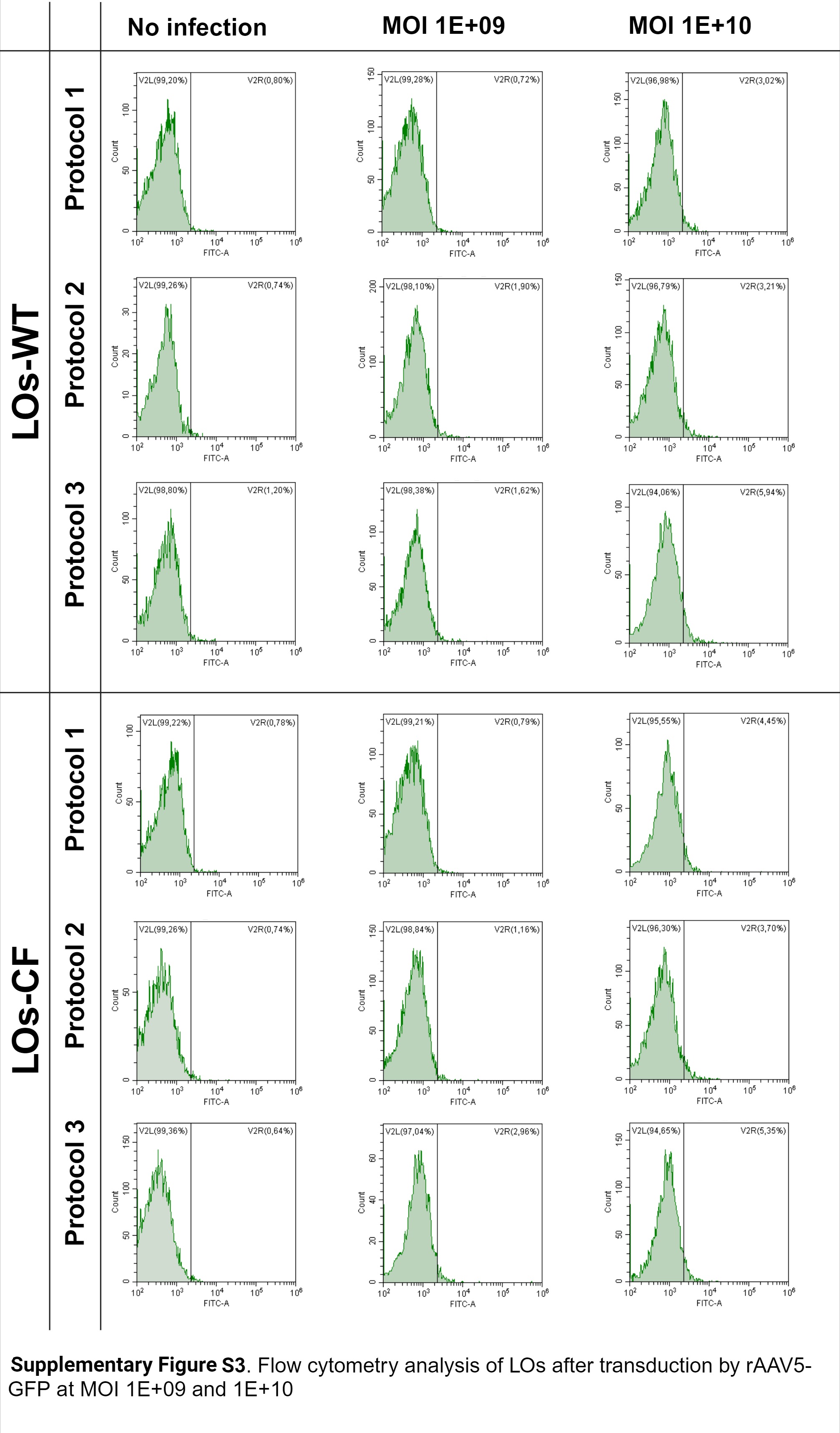

Supplement: Supplementary file 1 [file biomedicines-13-00879-s001.zip › Supplementary Figure S3.jpeg]

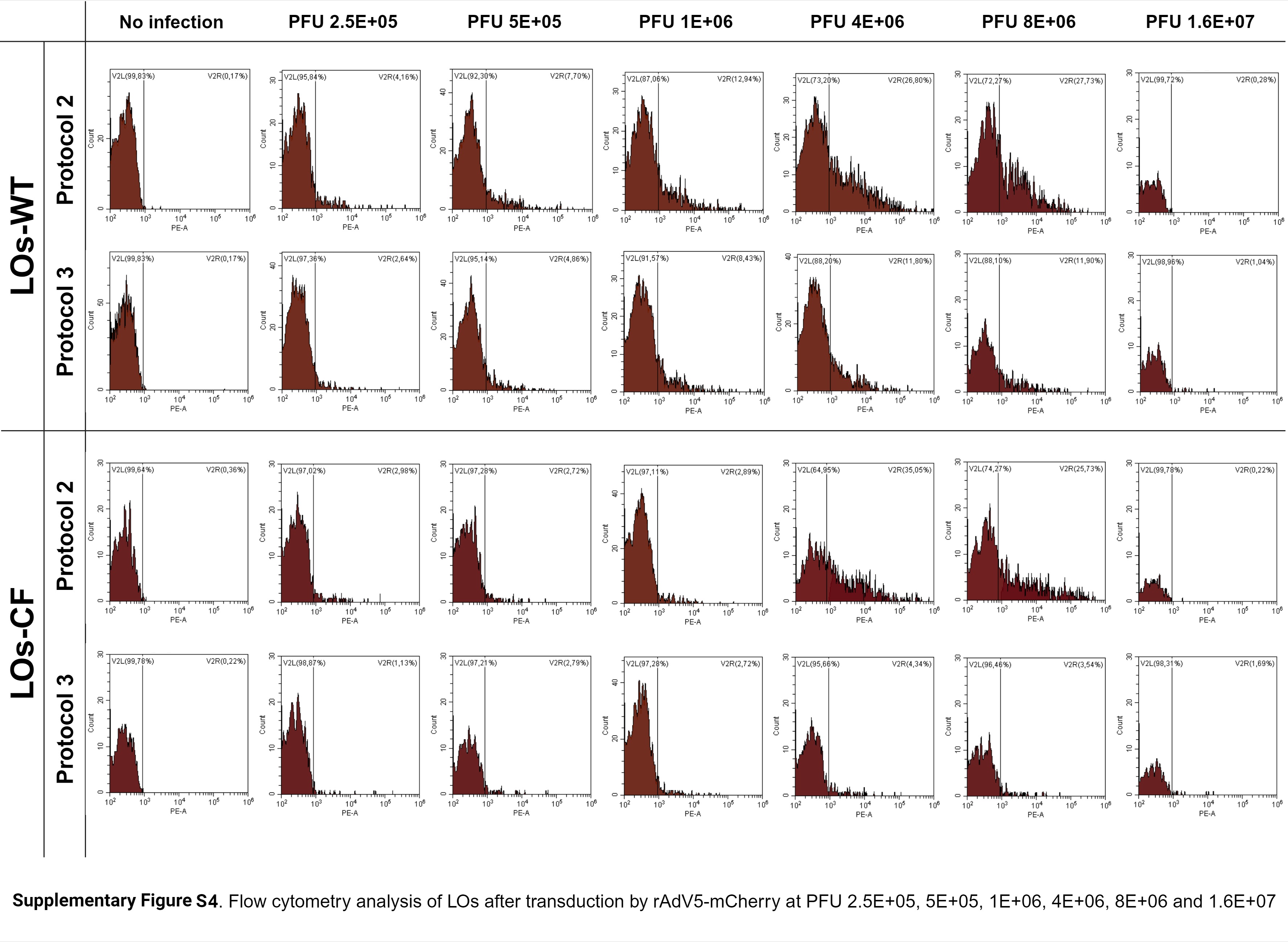

Supplement: Supplementary file 1 [file biomedicines-13-00879-s001.zip › Supplementary Figure S4.jpeg]

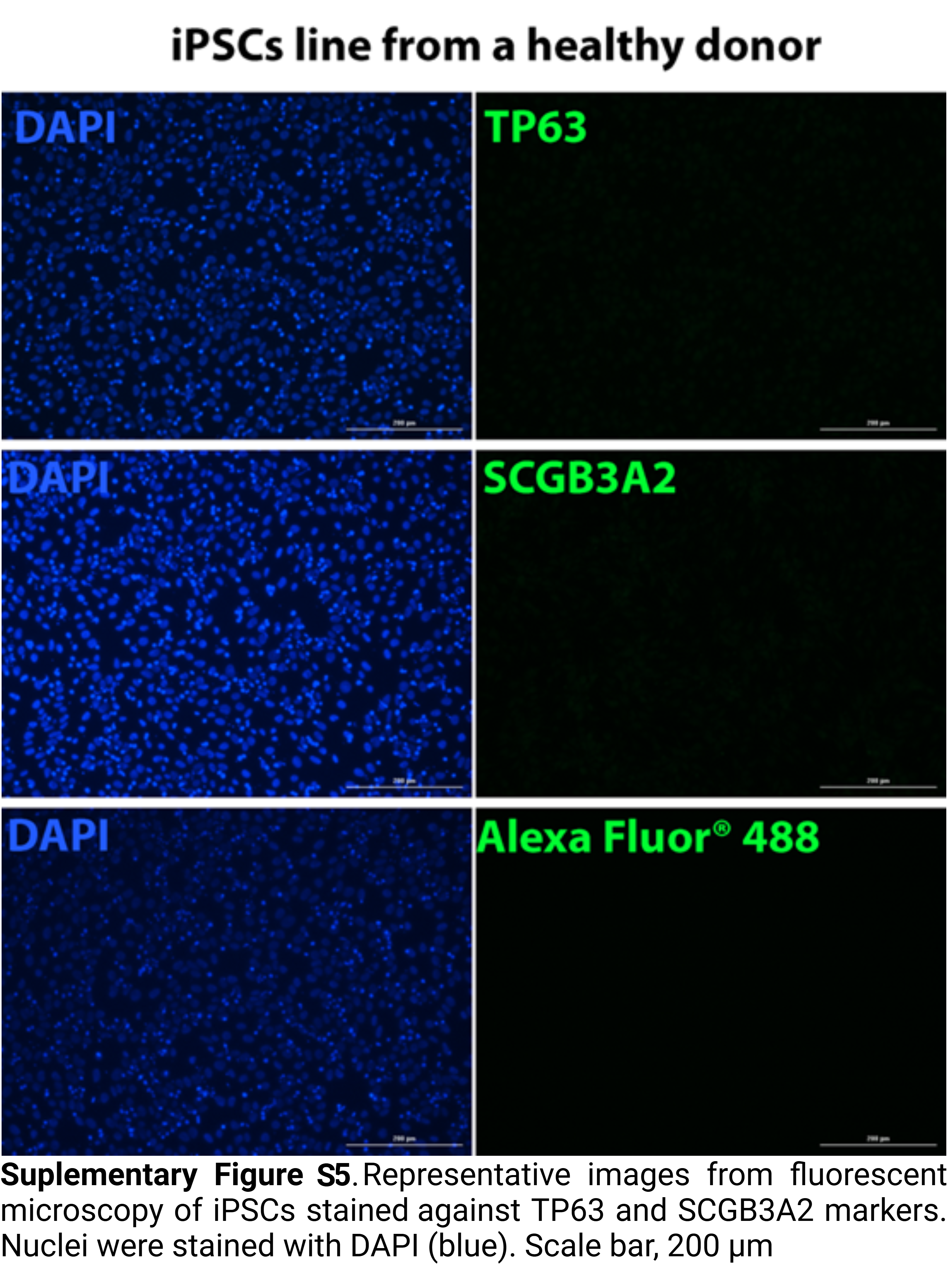

Supplement: Supplementary file 1 [file biomedicines-13-00879-s001.zip › Supplementary Figure S5.png]

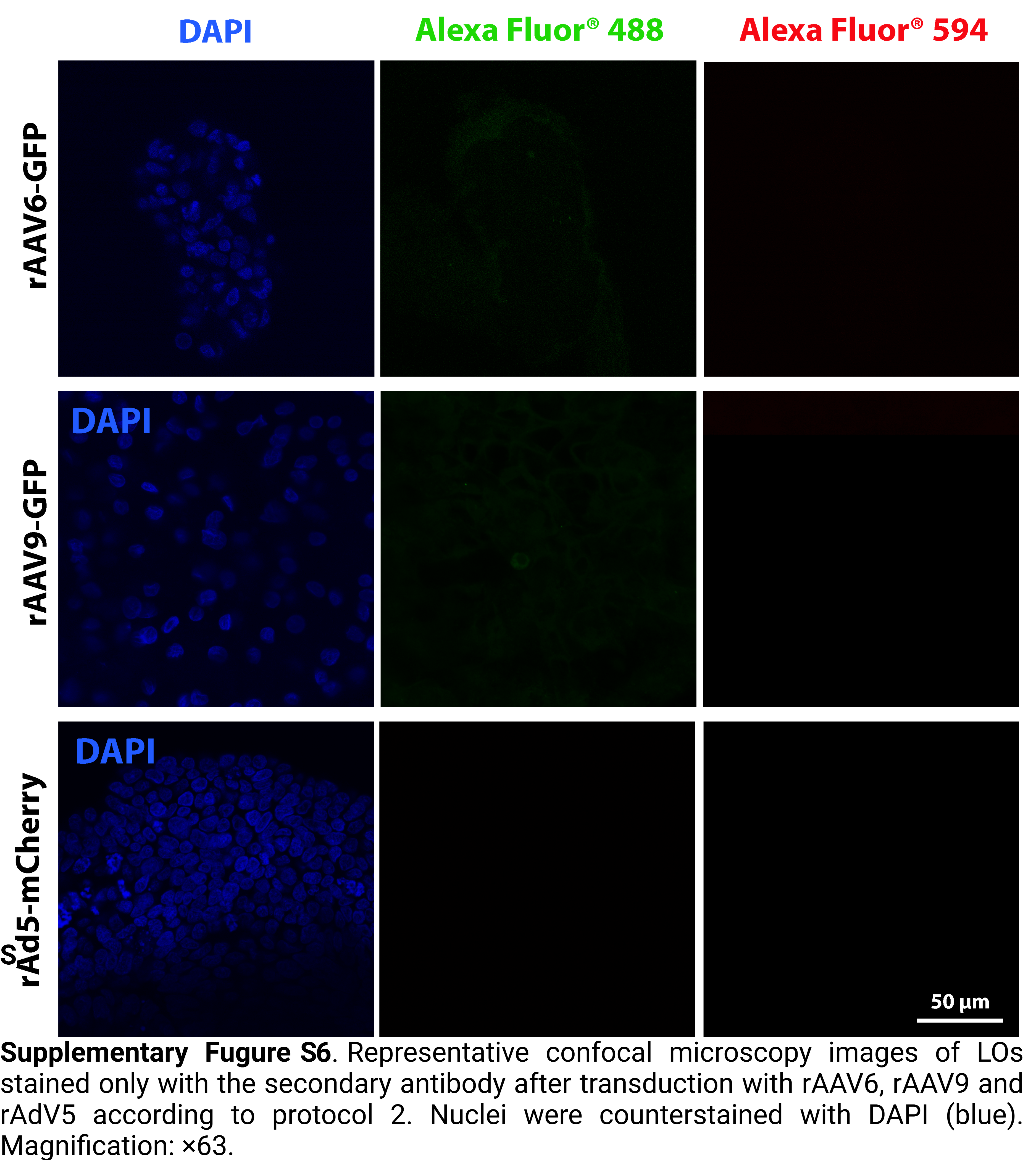

Supplement: Supplementary file 1 [file biomedicines-13-00879-s001.zip › Supplementary Figure S6.png]

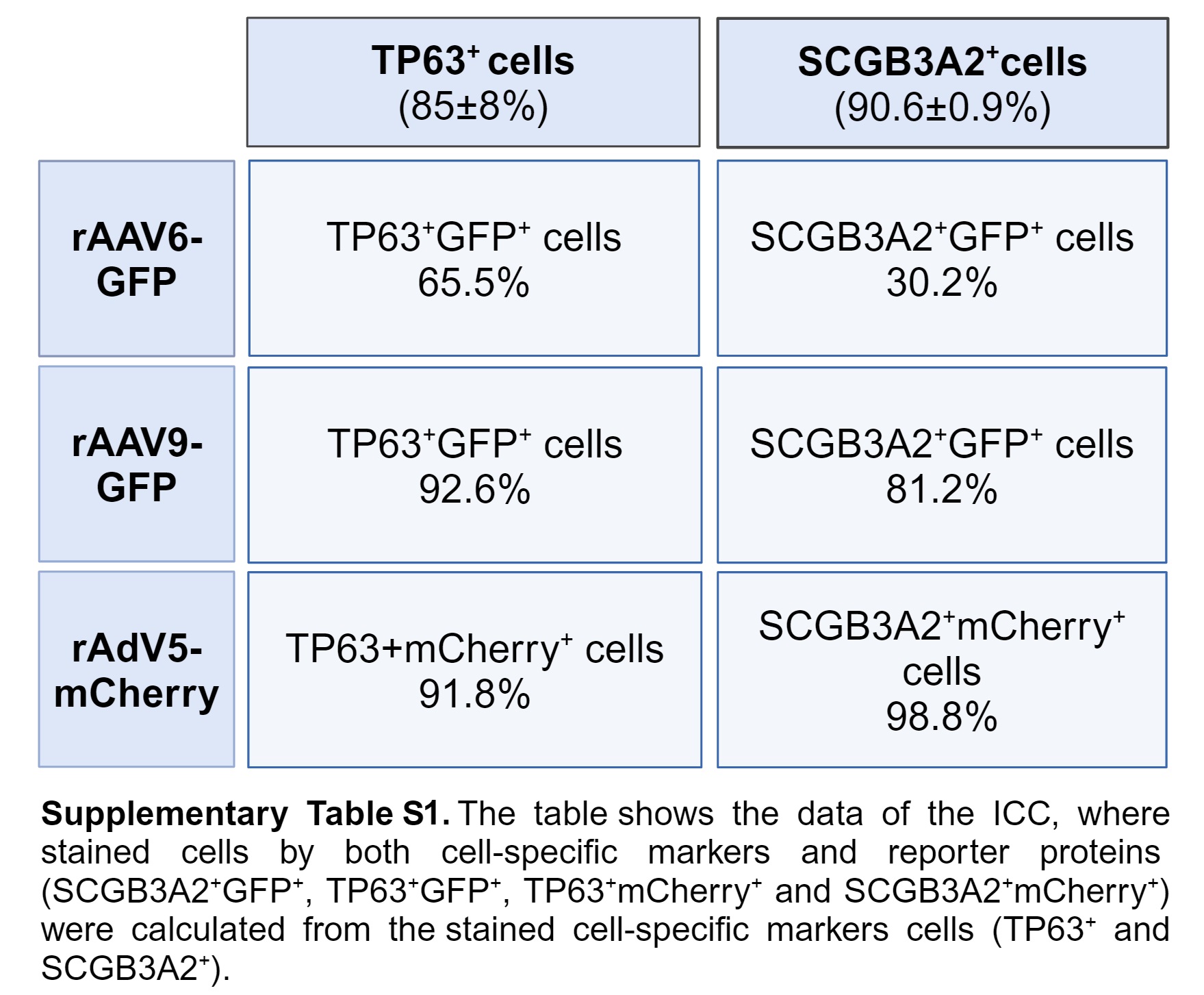

Supplement: Supplementary file 1 [file biomedicines-13-00879-s001.zip › Supplementary Table.jpeg]
